# Combined Endoscopic Laser and Cryotherapy Management of Pediatric Central Airway Obstruction Secondary to a Histoplasmosis-Related Broncholith

**DOI:** 10.7759/cureus.45492

**Published:** 2023-09-18

**Authors:** Abigail E Moore, Malak J Gazzaz, Rolando Sanchez, Sohit P Kanotra

**Affiliations:** 1 Otolaryngology - Head and Neck Surgery, University of Iowa Hospitals and Clinics, Iowa City, USA; 2 Otolaryngology - Head and Neck Surgery, Umm AlQura University, Makkah, SAU; 3 Pulmonary and Critical Care Medicine, University of Iowa Hospitals and Clinics, Iowa City, USA; 4 Otolaryngology, University of Iowa Hospitals and Clinics, Iowa City, USA

**Keywords:** histioplasmosis, pediatric endoscopy, airway disorders, laser treatment, broncholithiasis

## Abstract

Broncholithiasis due to pulmonary histoplasmosis causing central airway obstruction and broncho-mediastinal fistula is a rare complication in the pediatric population. A 16-year-old previously healthy female was referred to a university hospital for worsening cough and shortness of breath for over a two-year period. Radiologic investigation revealed a calcified subcarinal lymph node eroding into the left mainstem bronchus causing central airway obstruction and collapse of the left lower lobe. Direct laryngoscopy and bronchoscopy showed a large obstructive lesion in the left mainstem bronchus. Debulking of the endobronchial lesion was performed with neodymium-doped yttrium aluminum garnet (Nd:YAG) laser and cryotherapy. Pathology examination was consistent with broncholith. Great clinical and radiological response to the procedure was evident with complete re-expansion of the left lung and resolution of symptoms.

## Introduction

Broncholithiasis is the presence of calcified material within the bronchial tree causing inflammation or obstruction [1]. Broncholithiasis is rare in pediatric populations with only a few reported cases [2]. Treatment depends on the type and severity of complications associated with it, and it usually requires a multidisciplinary approach. We describe a case of a 16-year-old female who presented with a broncholith, related to previous pulmonary histoplasmosis causing broncho-mediastinal fistula and complete central airway obstruction with lung collapse. This was successfully treated with combined neodymium-doped yttrium aluminum garnet (Nd:YAG) laser, cryotherapy, forceps, and electrocautery. A two-year follow-up did not show any evidence of recurrence. 

## Case presentation

**Figure 1:**
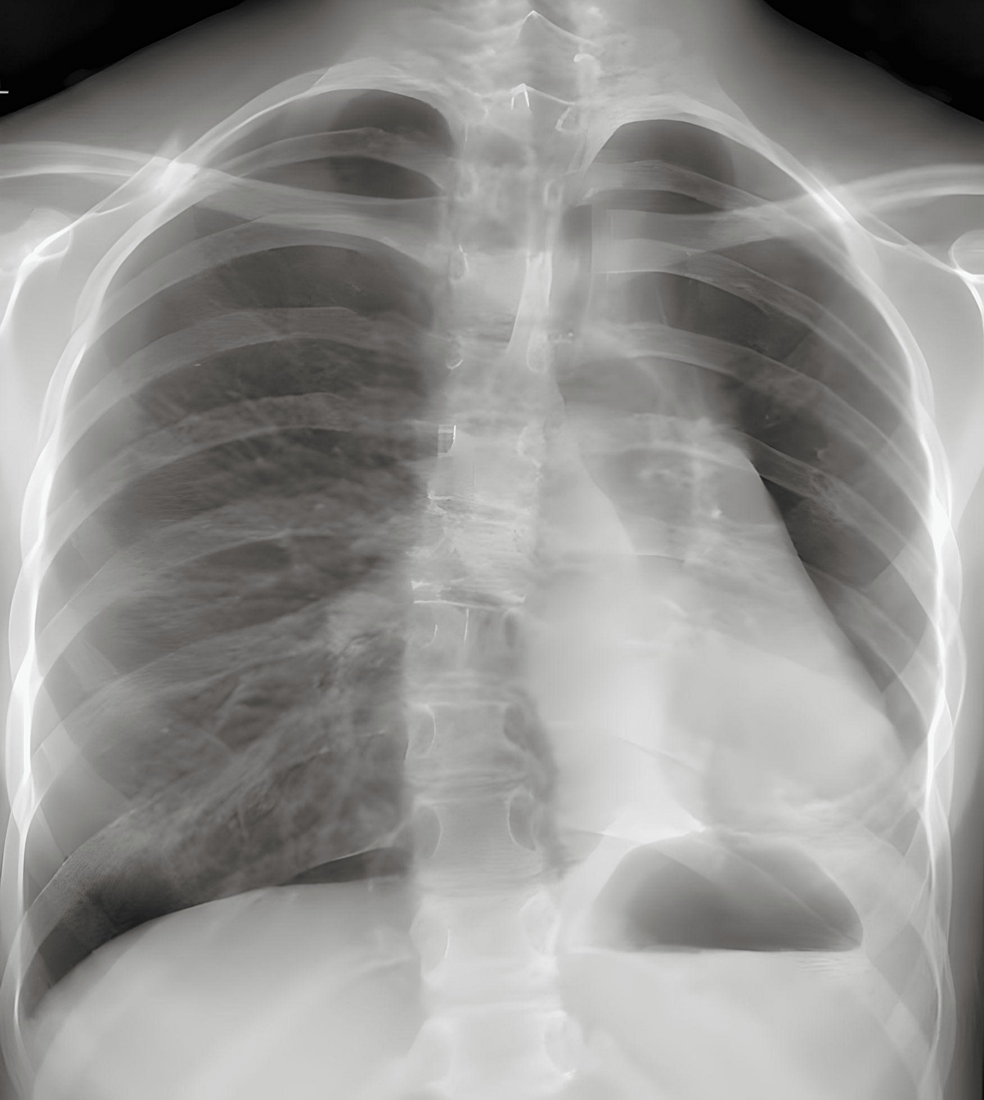
Chest X-ray showing hyperinflation of the right lung. Left retrocardiac consolidation or atelectasis. There is the elevation of the left hemidiaphragm. The cardiomediastinal silhouette is deviated to the left.

**Figure 2:**
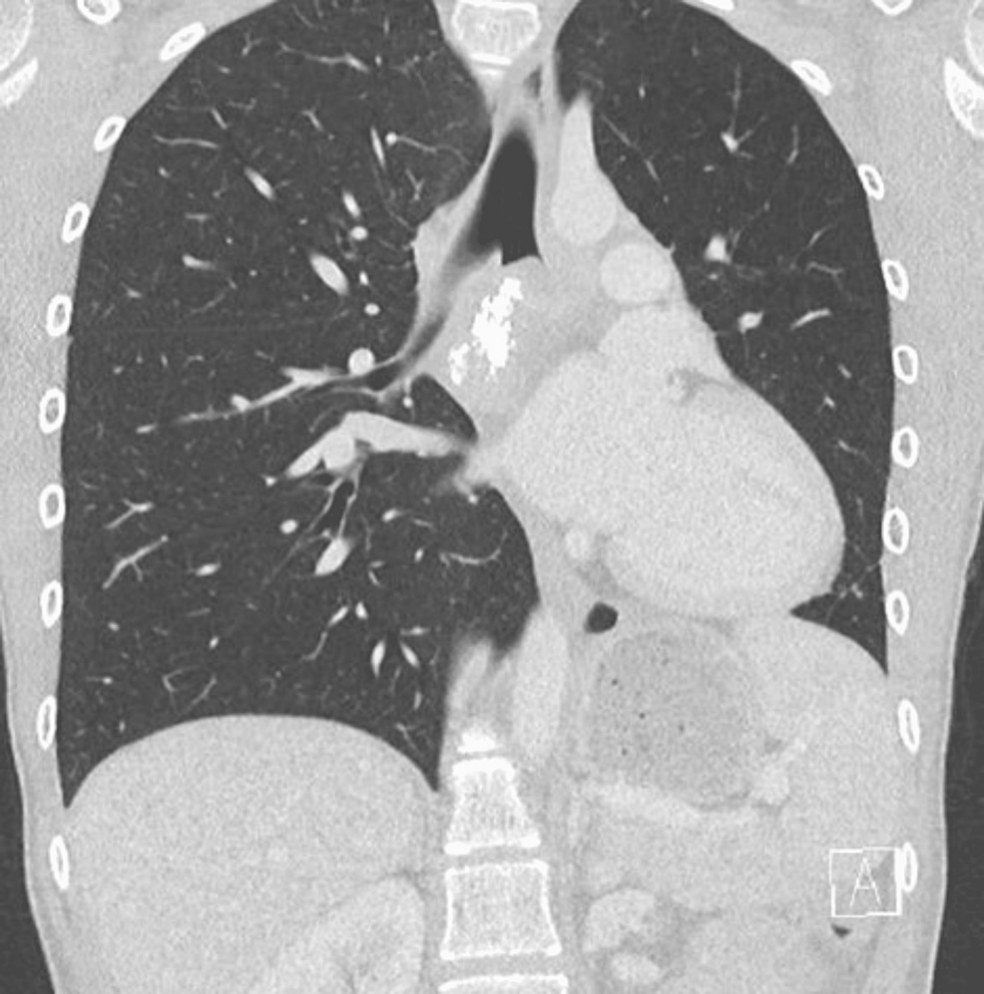
Chest CT coronal axial lung window views demonstrating subcarinal calcified lymph nodes with opacification and obstruction of the left mainstem bronchus. One of these calcifications is eroding the medial wall of the left mainstem bronchus. CT, computed tomography.

A 16-year-old female was evaluated for persistent dyspnea on exertion, cough, and wheezing, which had been ongoing for two years. She was being managed with inhaled steroids (Flovent 88 micrograms (mcg), 2 puffs twice daily) by the pediatrician. Her past medical history was insignificant for any factors contributing to chronic lung disease. Her physical examination was normal except for the presence of bilateral basal crackles. Pulmonary function test (PFT) showed mild lower airway obstruction with air trapping without bronchodilator reversibility (forced vital capacity (FVC) = 2.06 L, forced expiratory volume in the first second (FEV1) = 1.47 L, FEV1/FVC = 71%, total lung capacity (TLC) = 3.24, residual volume (RV) = 1.50). A chest X-ray (CXR) (Figure 1) revealed left lung collapse prompting a computed tomography (CT) (Figure 2), which showed multiple calcified subcarinal and left hilar lymph nodes with mass effect surrounding the subcarinal lymph node extending into the left main bronchus with opacification of the distal left main airway associated with partial collapse of the left lower lobe. Laboratory workup included complete blood count (CBC), C-reactive protein (CRP), blood and urine testing for histoplasma antigen, and purified protein derivative (PPD), which were all within normal limits. After discussion in a multidisciplinary airway conference, the decision was made to proceed with a multimodality treatment for definitive management. A preoperative course of prednisone, 40 milligrams (mg) twice daily for seven days, was started. Itraconazole 200 mg twice daily was initiated for empiric treatment of pulmonary histoplasmosis.

**Figure 3:**
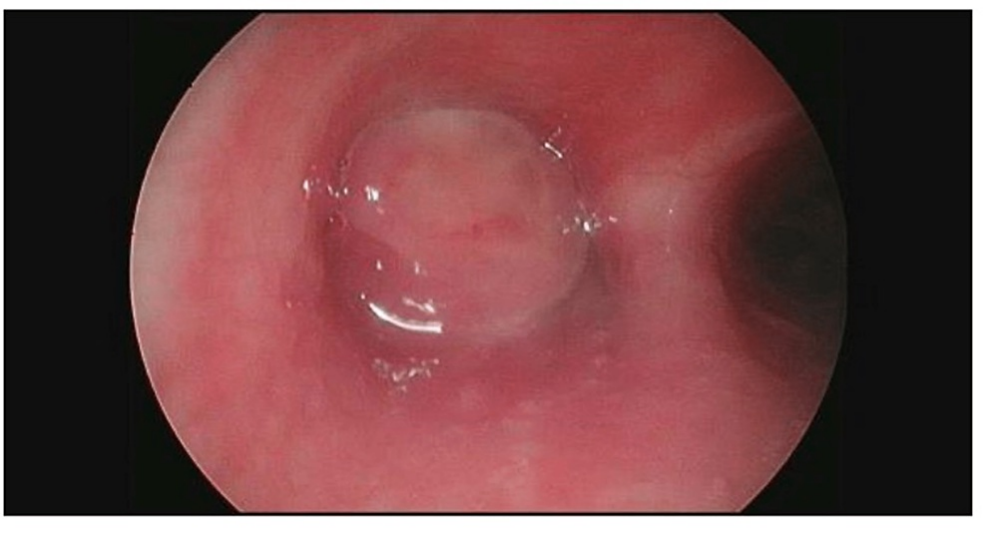
A large, completely obstructive lesion of the proximal left mainstem bronchus seen on rigid bronchoscopy.

**Figure 4:**
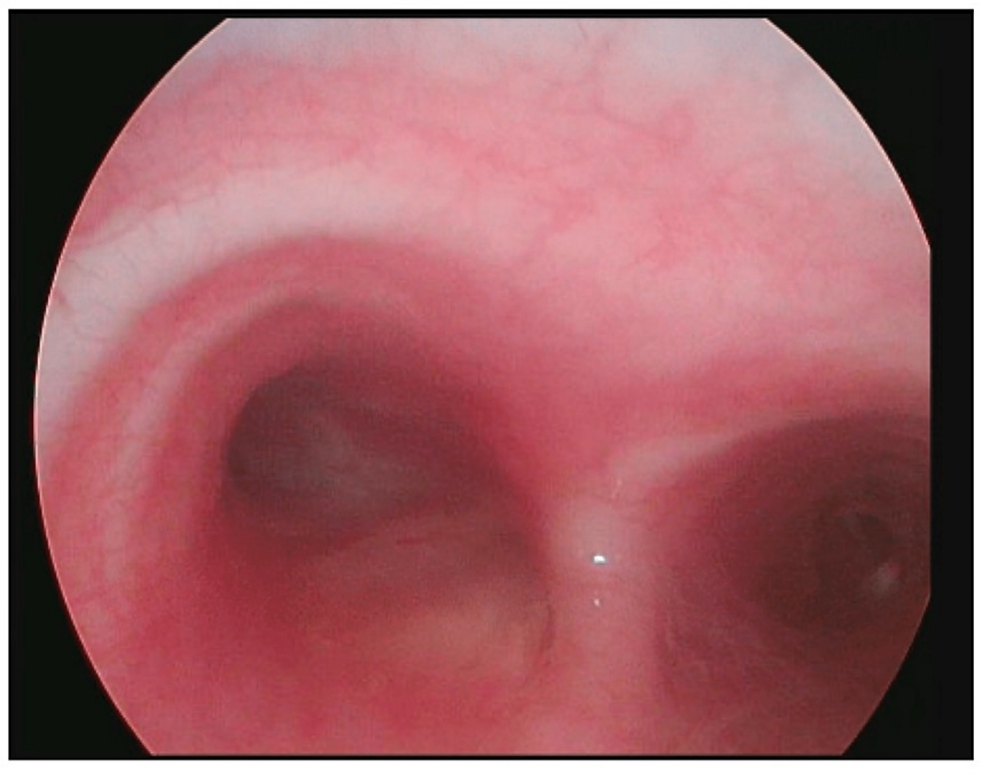
Left mainstem medial wall with a well-healing site of prior lesion debulking on rigid bronchoscopy.

Diagnostic rigid bronchoscopy in the operating room (OR) showed an occlusive mass completely blocking the left mainstem bronchus with surrounding granulation that appeared to be originating and communicating with the subcarinal broncholith (Figure 3). Postoperatively, she was given three doses of dexamethasone 6 mg intravenously (IV) at 6-hour intervals. A planned therapeutic suspension laryngoscopy and rigid bronchoscopy were performed in the OR under general anesthesia by pediatric otolaryngology and interventional pulmonology. Airway inspection showed an obstructive, yet slightly smaller lesion in the left mainstem bronchus. An Nd:YAG laser fiber was taped to a zero-degree Hopkins rod endoscope to gain access to the lesion. It was set at 40 watts, a frequency of 4 Hz, and was first utilized to reduce the size of granulation tissue and provide hemostasis. Granulation tissue was significantly debulked with minimal bleeding. Next, a flexible therapeutic bronchoscope (Olympus T190, Olympus America, Center Valley, Pennsylvania, United States) was used to debulk the broncholith using cryotherapy and forceps. Samples obtained were sent to pathology for further examination. The obstructive mass was completely removed with minimal bleeding which was controlled with electrocautery. Postoperatively, she was given three doses of dexamethasone 6 mg IV at 6-hour intervals and clindamycin 300 mg orally three times a day for one week for prevention of suppurative mediastinitis given the communication between the left bronchus and the mediastinum. Anatomic pathology examination of the biopsies showed inflamed granulation tissue and calcified amorphous material compatible with a broncholith. Special staining including Gömöri Methenamine Silver (GMS) was negative for microorganisms. Postoperative bronchoscopy revealed a completely healed left mainstem bronchus with no evidence of any occlusion (Figure 4).

The patient completed a total of six months of itraconazole therapy. At the six-month follow-up, she had a complete resolution of symptoms and her PFT normalized. 

Anatomic pathology examination of the biopsies showed inflamed granulation tissue and calcified amorphous material compatible with a broncholith. Special staining including GMS was negative for microorganisms. Postoperative bronchoscopy revealed a completely healed left mainstem bronchus with no evidence of any occlusion (Figure 4). The patient completed a total of six months of itraconazole therapy. At the six-month follow-up, she had a complete resolution of symptoms and her PFT normalized. A two-year follow-up did not reveal any recurrence.

## Discussion

We report a case of histoplasmosis-related broncholithiasis with erosion into the bronchial wall (broncho-mediastinal fistula) causing obstruction of the left mainstem bronchus in an immunocompetent pediatric patient. The surgical management was challenging in this case considering the accessibility of the lesion. The management of this case highlights important points including the use of combined modality. The use of Nd:YAG laser in the setting of a vascular lesion in the bronchus provided adequate hemostasis. The use of cryotherapy helped in the complete removal of obstruction after initial debulking with a laser.

Broncholithiasis results from granulomatous inflammation and calcification of mediastinal and/or hilar lymph nodes, secondary to infection by atypical organisms (tuberculosis or endemic fungal infection) or chronic granulomatous diseases like sarcoidosis, which erode through the bronchial wall. Broncholithiasis can be asymptomatic or can cause complications like airway inflammation and remodeling, airway obstruction with atelectasis, recurrent pneumonia, dyspnea, cough, hemoptysis, and mediastinitis from broncho-mediastinal fistula formation [3]. Pulmonary histoplasmosis is the most common cause of broncholithiasis in endemic areas [4]. The clinical presentation of pulmonary histoplasmosis infection depends on the size of the pathogen inoculum, and the immune response by the host. The disseminated disease usually occurs with very large inoculums and/or in immunosuppressed patients. The infection is usually self-limited and is asymptomatic in immunocompetent patients with small inoculums. Some patients can develop severe granulomatous inflammation in the lung and mediastinum in reaction to the fungal antigens. Broncholithiasis represents the mild spectrum of mediastinal fibrosis, while fibrosing mediastinitis represents the most severe form and it is usually associated with life-threatening complications from the compression of mediastinal organs and the main vessels [4]. Diagnosis can be difficult and is usually delayed because the symptoms often resemble other, more common, airway diseases such as asthma, as initially mistaken in this case. The diagnosis of pulmonary histoplasmosis in our patient was based on the clinical presentation, epidemiological exposure (residence in an endemic area), and excluding other causes of chronic granulomatous diseases. Since broncholithiasis and fibrosing mediastinitis are usually late complications of pulmonary histoplasmosis, the yield of histoplasma serologies and histoplasma antigens in the urine and blood are very low in these cases. Pathology examination usually shows granulomatous inflammation but the isolation of the fungus in tissue or cultures is rare, as exemplified by our case.

The management of broncholithiasis depends on the clinical presentation. Asymptomatic patients can often be managed conservatively through observation. There is also a limited role for conservative management such as bronchodilators and cough suppression in those who have minimal symptoms or significant comorbidities [5]. Empiric antimicrobial therapy may be used in suspected superimposed bacterial pneumonia. There is no proven benefit of antifungal and systemic steroid therapy; however, both are commonly used in real practice [6]. Those with airway obstruction or severe symptoms, such as recurrent pneumonia or massive hemoptysis, will often require surgical intervention [7]. It has been recently recommended that bronchoscopy be attempted before pursuing more invasive surgical approaches [8]. Thoracotomy may be required after failure of therapeutic bronchoscopy, firmly embedded broncholiths, involvement of other mediastinal structures, or massive hemoptysis. It is associated with a higher potential for lung function compromise than bronchoscopy due to the possibility of lung parenchyma destruction [1].

Our patient was treated successfully with a combination of Nd:YAG laser and cryotherapy allowing for immediate relief of airway obstruction and associated symptoms, without causing significant scarring of the bronchial mucosa. Broncholithiasis causing airway obstruction is rare in pediatric patients. In 2012, Lee et al. described the first case of pediatric broncholithiasis caused by histoplasmosis in an immunocompetent 15-year-old [2].

Flexible or rigid bronchoscopy can be used both in the diagnosis and treatment of broncholithiasis. Free or minimally adhered stones can be removed with forceps or a balloon catheter during bronchoscopy. This technique when used should be done with extreme caution in the removal of a fixed broncholith due to the risk of significant bleeding or airway wall trauma [9]. Large size, difficult location, or presence of significant amount of granulation tissue may also limit the use of this method. Recently, the use of Nd:YAG and holmium:neodymium-yttrium, aluminum, garnet (Ho:YAG) laser lithotripsy for large or transbronchial broncholiths has been described, both with a high treatment success rate [10,11]. Both laser types have good coagulation properties, which minimizes the amount of bleeding encountered. The biggest risks in the use of laser therapy are airway damage in the form of perforation and the risk of airway fire. The fraction of inspired oxygen (FiO_2_) and power/frequency settings should be carefully chosen [11].

The use of Nd:YAG laser was favorable in our patient because it was involving a mainstem bronchus, the lesion was exophytic, and the patient was hemodynamically stable and was able to tolerate FiO_2_ less than 40% during laser therapy [10]. We used a noncontact laser, which eliminated the risk of serious side effects such as air embolism and pneumomediastinum. The use of systemic steroids may have helped reduce the amount of granulation tissue before debulking in our patient.

Cryotherapy has also been described for the treatment of granulation tissue. However, cryotherapy is not useful in the removal of broncholith because of its low water content. Calcification prevents it from being frozen easily. Advantages of debulking granulation tissue in the airway using cryotherapy include the ability to maintain high FiO_2_, ease of use, lower cost, and less likelihood to cause scarring. The major downside is the inadequate hemostatic property [12,13]. We elected to use both laser therapy and cryotherapy to compensate for the disadvantages of each method and thus obtain better results. The two modalities should be considered after careful examination of chest imaging and the relationship to the surrounding vascular structure to avoid catastrophic bleeds [11,12].

Our experience with this patient indicates that a combination of cryotherapy and laser is a reasonable treatment option for the removal of an obstructive broncholith with the combination of rigid and flexible bronchoscopy.

## Conclusions

Broncholithiasis is a rare presentation in pediatric patients and diagnosis may be difficult due to non-specific symptoms that overlap with other common airway disorders. Management of airway obstruction caused by broncholithiasis depends on the severity of presentation, and broncholith’s size, mobility, and location. A multidisciplinary approach is usually required for the treatment of airway obstruction secondary to broncholithiasis. A combination of Nd:YAG laser and cryotherapy is a feasible treatment option.
